# Soybean transcription factor ORFeome associated with drought resistance: a valuable resource to accelerate research on abiotic stress resistance

**DOI:** 10.1186/s12864-015-1743-6

**Published:** 2015-08-13

**Authors:** Chenglin Chai, Yongqin Wang, Trupti Joshi, Babu Valliyodan, Silvas Prince, Lydia Michel, Dong Xu, Henry T. Nguyen

**Affiliations:** Division of Plant Sciences and National Center for Soybean Biotechnology, University of Missouri, Columbia, MO 65211 USA; Department of Computer Science, Informatics Institute, Christopher S. Bond Life Sciences Center, University of Missouri, Columbia, MO 65211 USA

**Keywords:** Soybean, Transcription factor, ORFeome, Abiotic stresses, Drought, Salinity, Dehydration, ABA, *cis*-regulatory element

## Abstract

**Background:**

Whole genome sequencing provides the most comprehensive collection of an organism’s genetic information. The availability of complete genome sequences is expected to dramatically deliver a high impact on biology. However, to achieve this impact in the area of crop improvement, significant efforts are still required on functional genomics, including the areas of gene annotation, cloning, expression profiling, and functional validation.

**Results:**

Here we report our efforts in generating the first transcription factor (TF) open reading frame (ORF)eome resource associated with drought resistance in soybean (*Glycine max*), a major oil/protein crop grown worldwide. This study provides a highly annotated soybean TF-ORFeome associated with drought resistance. It contains information from experimentally verified protein-coding sequences (CDS), expression profiling under several abiotic stresses (drought, salinity, dehydration and ABA), and computationally predicted protein subcellular localization and *cis*-regulatory elements (CREs) analysis. All the information is available to plant researchers through a freely accessible and user-friendly database, Soybean Knowledge Base (SoyKB).

**Conclusions:**

The soybean TF-ORFeome provides a valuable public resource for functional genomics studies, especially in the area of plant abiotic stresses. It will accelerate findings in the areas of abiotic stresses and lead to the generation of crops with enhanced resistance to multiple stresses.

**Electronic supplementary material:**

The online version of this article (doi:10.1186/s12864-015-1743-6) contains supplementary material, which is available to authorized users.

## Background

Whole genome sequencing (WGS) provides the most comprehensive collection of an organism’s genetic information. Large-scale genome sequencing is expected to change the way in which biology has traditionally been conducted. The ever-decreasing cost of sequencing is moving towards a new era in plant genetic and genomic studies. By taking advantage of large data acquisition platforms, genomes from more than 40 plants of agronomical importance have been sequenced so far [1]. However, to achieve this promise of WGS in research focused on crop improvement, significant efforts are required in functional genomics that include gene annotation, cloning, expression, and further functional analysis.

Knowledge of gene sequences and of the deduced protein sequences is very important in determining protein functions. In this process, large genomic resources such as expressed sequence tag (EST) databases, full-length complimentary DNA (cDNA) libraries, and open reading frame (ORF) collections (ORFeome) have played important roles. Although EST databases and computational predictions are useful, the EST databases usually provide only partial transcribed sequences that could be misleading, while the automated computational predication are not fully accurate [2]. Full-length cDNA libraries contain full-ORFs plus 5′ and 3′ un-translated regions (UTRs), which will allow massive functional screening in various fields of biology. However, the drawback of cDNA libraries has become obvious due to the interference of 5′- and 3′- UTRs, and low coverage of cDNA libraries for total gene transcripts [3]. ORFeome collections not only overcome the problems mentioned above, but also have additional advantages. By using gene-specific primers, genuine full ORFs can be obtained, which assure high coverage and no interference of 5′- or 3′- UTRs. The recombination-based cloning techniques including Gateway cloning [4], have revolutionized the ways of conventional “cut-and-paste” techniques, and greatly expedited high-throughput gene cloning. Furthermore, access to the ORF cDNA clones would facilitate various functional studies of genes and corresponding proteins by transferring ORFs via LR reactions from Entry clones into Gateway-compatible expression vectors [5]. ORFeome resources have been successfully applied in genome annotation, genome-wide protein localization, metabolic structure studies, proteomics, comparative functional genomics, global mapping protein-protein interaction and DNA-protein interactions [6-11]. However, despite all the achievements made so far by plant scientists in building various ORFeomic resources, most existing ORFeomes are too general. This leads to a situation wherein researchers working in a specific area (e.g., drought research) have to spend a significant amount of time finding information in their area of interest.

Soybean (*Glycine max*) is the most important cash crop widely grown for its high protein and oil content, beneficial phytochemicals, and production as biodiesel. However, its growth and grain yield are highly affected by soil water availability. Drought stresses have caused significant yield losses worldwide [12, 13]. Plants respond and adapt to drought stress conditions with an array of molecular, biochemical, and physiological alterations. Despite the fact that the soybean’s entire genome was sequenced several years ago [14], the exact transcript structures of the majority of its protein-coding genes remain experimentally unverified. As such, there is an urgent need in the soybean community for ORFeome clones of protein-coding genes. Since TFs are master regulators in controlling many, if not all, of the biological processes such as development, growth, cell division, and responses to environmental stimuli, our efforts in this study are focused on generating the first transcription factor (TF) ORFeome resource associated with drought resistance in soybean. The soybean TF-ORFeome related information has been deposited in the Soybean Knowledge Base (SoyKB) [15-17] and is available to the global research community for comprehensive functional characterization. This will greatly accelerate findings in the area of drought resistance research.

## Results and discussion

### Soybean TF selection and cloning

**Fig. 1:**
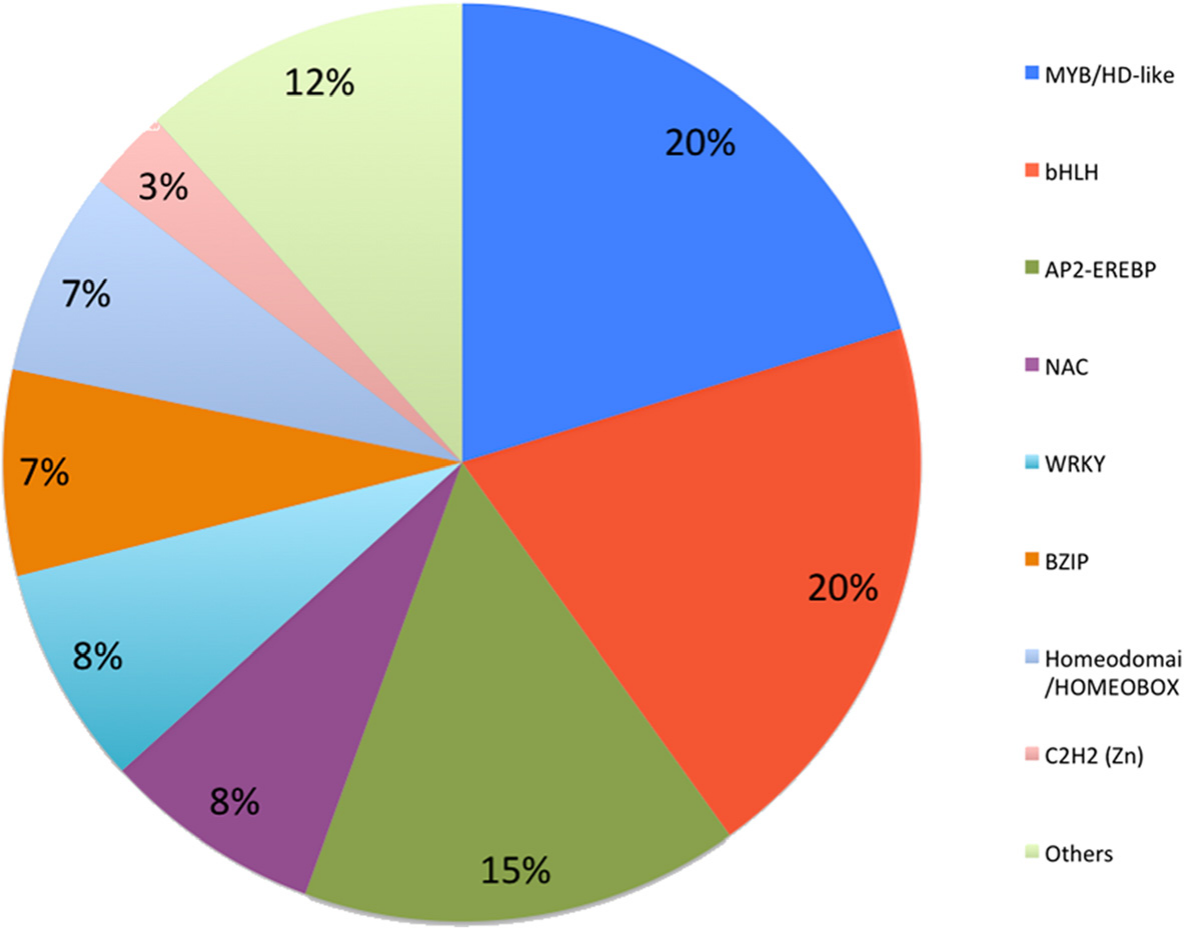
Distribution of cloned soybean TF-ORFs among different gene families

Mainly based on microarray data of soybean root and leaf under dehydration and drought conditions generated by our group (Valliyodan et al., unpublished data) and other researchers [18], soybean TFs with a fold change ≥ 1.5 upon treatments and a *p*-value <0.05 were selected as candidates for building this soybean TF-ORFeome (Additional file 1). We also included in this TF-ORFeome 19 TFs, which showed a fold change ≥1.5 in at least one of the two tissues (shoots and roots) upon mild drought stress in our quantitative reverse transcription-PCR (qRT-PCR) analysis (Additional file 2A) but without support from our microarray data due to lack of probes [18]. A total of 207 soybean full-length TF ORFs were cloned into pENTR™/D TOPO or pDONR™/Zeo vectors, which meets the “gold standard” criteria as previously defined [3]. Detailed information of these clones is provided (Additional file 3), including gene locus number, transcript, GenBank accession number, gene family, gene size, vector, ORF sequence, primer sequences, with/without stop codon, and others. These TFs were not equally distributed among 21 gene families, of which the top seven families are MYB, bHLH, APETALA2 (AP2)-ethylene-responsive element binding protein (EREBP), NAC, WRKY, bZIP and Cys2(C2)His2(H2)-type zinc fingers (ZFs), constituting 88 % of the total clones (Fig. 1). Genes from these TF families were found to play important roles in responding to various abiotic stresses, which was very well summarized in a recent review paper [19]. Several genes from our ORFeome collection were reported as major regulators in the soybean abiotic stress responses, such as *GmbZIP1* (Glyma02g14880) [20], *GmERF3* (Glyma03g42450) [21], *GmDREB2* (Glyma06g04490) [22], and (*GmNAC004* (Glyma12g35000) [23]. However, functions of the vast majority soybean TFs are yet to be explored.

### Sequence analysis of cloned TFs

As expected, most (90.3 %) of the soybean TF ORFeome clones matched the gene annotation in the public database Phytozome (v 9.1) [24] based on sequencing results. For clones showing sequence differences, two independent RT-PCRs were performed to make certain that the sequence differences were not caused by errors during RT and/or PCR. At least two clones for each ORFeome were used for sequence verification. However, our sequence analysis revealed differences in 20 clones, 9.7 % of total TF-ORFs cloned in this study (Additional file 3). qRT-PCR analysis of expression changes of these genes upon mild drought stress treatment were conducted (Additional file 2B), and the results showed that most of them were positively regulated by the stress at the transcriptional level. The sequence differences might be due to alternative splicing, nucleotide replacement, insertion or deletion.

There are several possibilities for the sequence discrepancy in this study. Nearly 75 % of the soybean genes have paralogs, which were probably caused by two whole-genome duplication events that occurred between 59 and 13 million years ago, respectively [14]. Aligning the discrepant sequences back to the soybean genome excluded the possibility that they are one copy of the many duplicated genes, although it is still possible that the duplicated genes are located in the un-sequenced gaps. Another cause of sequence differences of the ORFs might be due to the genomic heterogeneity of Williams 82, which led to the intra-cultivar variations among individuals [25]. However, there is little chance of error from RT-PCR or sequencing due to the stringent conditions set for these processes and the use of multiple clones for sequence verification, as stated above.

### Expression profiles of selected TFs from ORFeome collection under drought, dehydration, salt and ABA conditions

Analysis of gene expression in different tissues and under different conditions is a useful way to predict gene functions. By searching the available whole genome profiling data, gene expression profiles of the TFs in 7 soybean tissues/organs (Additional file 4, data are from [26]) and under water deficit conditions (Additional file 1) were collected. Both the tissue expression patterns and the expression fold changes under water deficit conditions revealed a large amount of variation among different TFs, suggesting their diverse functions during soybean growth, development and adaption to water deficit conditions.

**Fig. 2:**
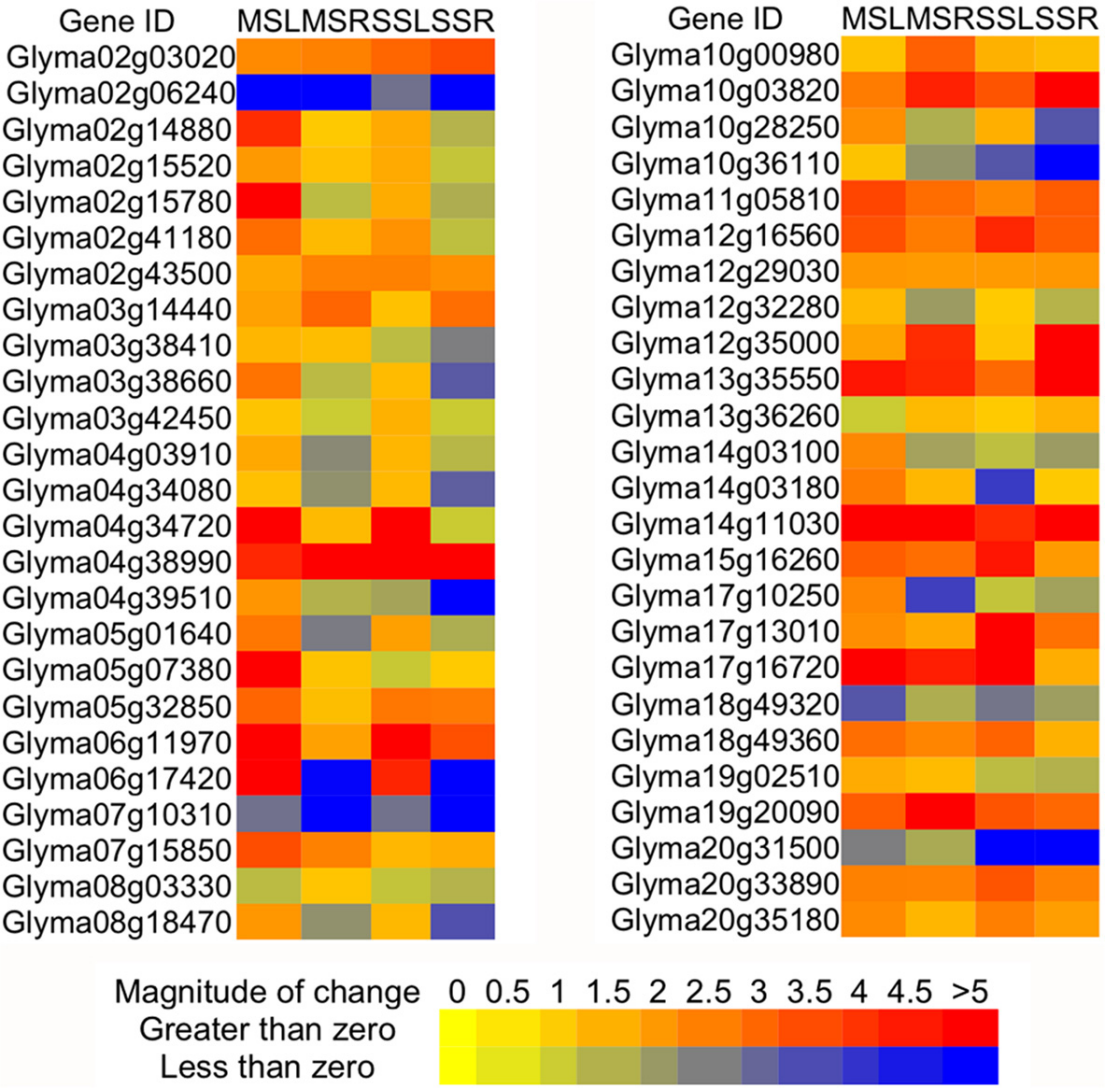
Expression of randomly selected 50 TFs under mild and moderate drought conditions. MSL, mild drought stress shoots; MSR, mild drought stress roots; SSL, moderate drought stress shoots; SSR, moderate drought stress roots

**Fig. 3:**
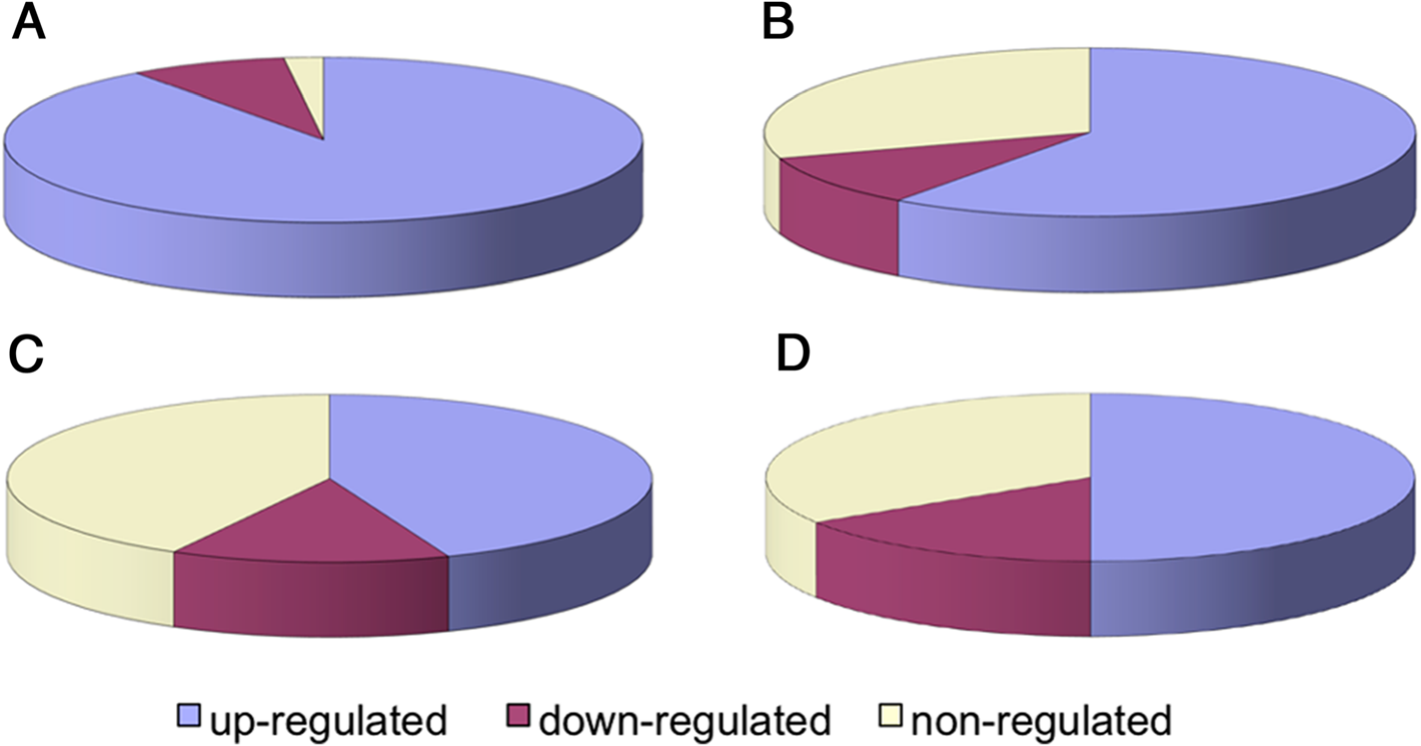
Soybean TFs up- and down-regulated in mild (**a** and **c**) and moderate (**b** and **d**) drought stresses. **a**, mild drought stress shoots; **b**, moderate drought stress shoots; **c**, mild drought stress roots; **d**, moderate drought stress roots

In order to provide more experimental support that the cloned TFs are responsive to water stress, expression profiles of 50 randomly selected soybean TFs (generated by the web tool: Research Randomizer [27]) were evaluated using qRT-PCR under conditions of drought, dehydration and salinity (Fig. 5). Upon drought treatments, 98 % of the selected genes were either up- or down-regulated in one or both of the drought conditions (Figs. 2 and 3). The total number of up- and down-regulated genes in roots was much smaller than in shoots under mild drought conditions (62 % vs. 98 %; Fig. 3a, c), while similar numbers of regulated genes were found under moderate stress conditions (72 % in roots vs. 70 % in shoots; Fig. 3b, d). The same TFs showing different expression levels upon drought treatments in different tissues suggested that they might have varying functions in each tissue in response to drought stress. Overall, our qRT-PCR data further confirmed that the TFs in our ORFeome collection were drought responsive.

**Fig. 4:**
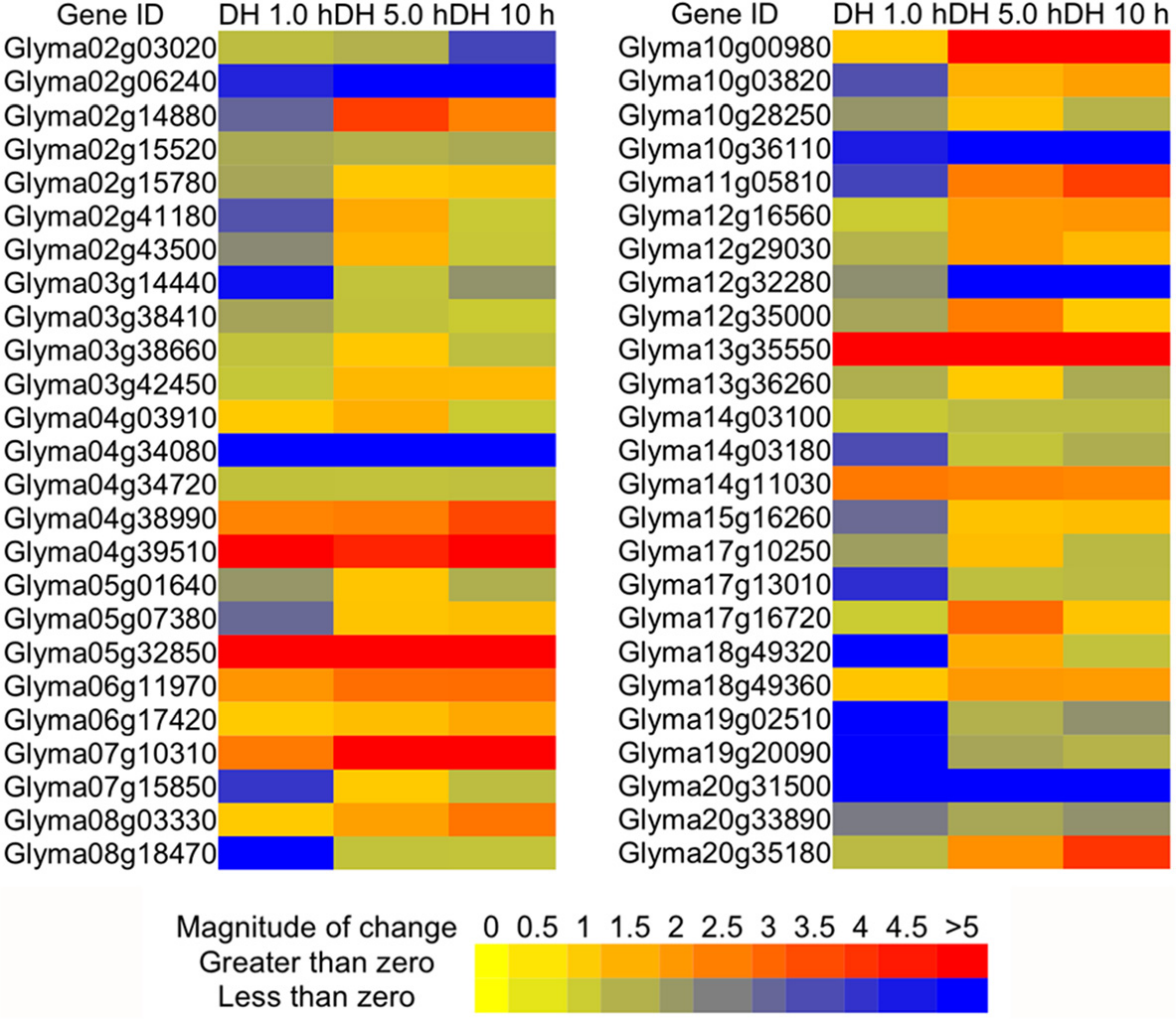
Expression of randomly selected 50 TFs under dehydration conditions for one hour (DH 1.0 h), five hours (DH 5.0 h) and ten hours (DH 10 h), respectively

Compared to drought, dehydration leads to a much lower water potential, and it is also considered as a common stress induced by drought, extreme temperature, or salinity conditions. Under dehydration treatments (Fig. 4), approximately 50 % of the selected genes were regulated the same way as in the drought conditions, while the expression patterns of other genes were quite different, indicating both pathways share some signaling components while remaining relatively independent. Notably, one GmNAC gene, Glyma12g35000.1, showed a dramatic up-regulation upon the dehydration treatment (75 fold change). A very recent study showed that over-expression of Glyma12g35000.1 in *Arabidopsis* enhanced lateral root-growth under both normal and mild drought stress conditions [23].

**Fig. 5:**
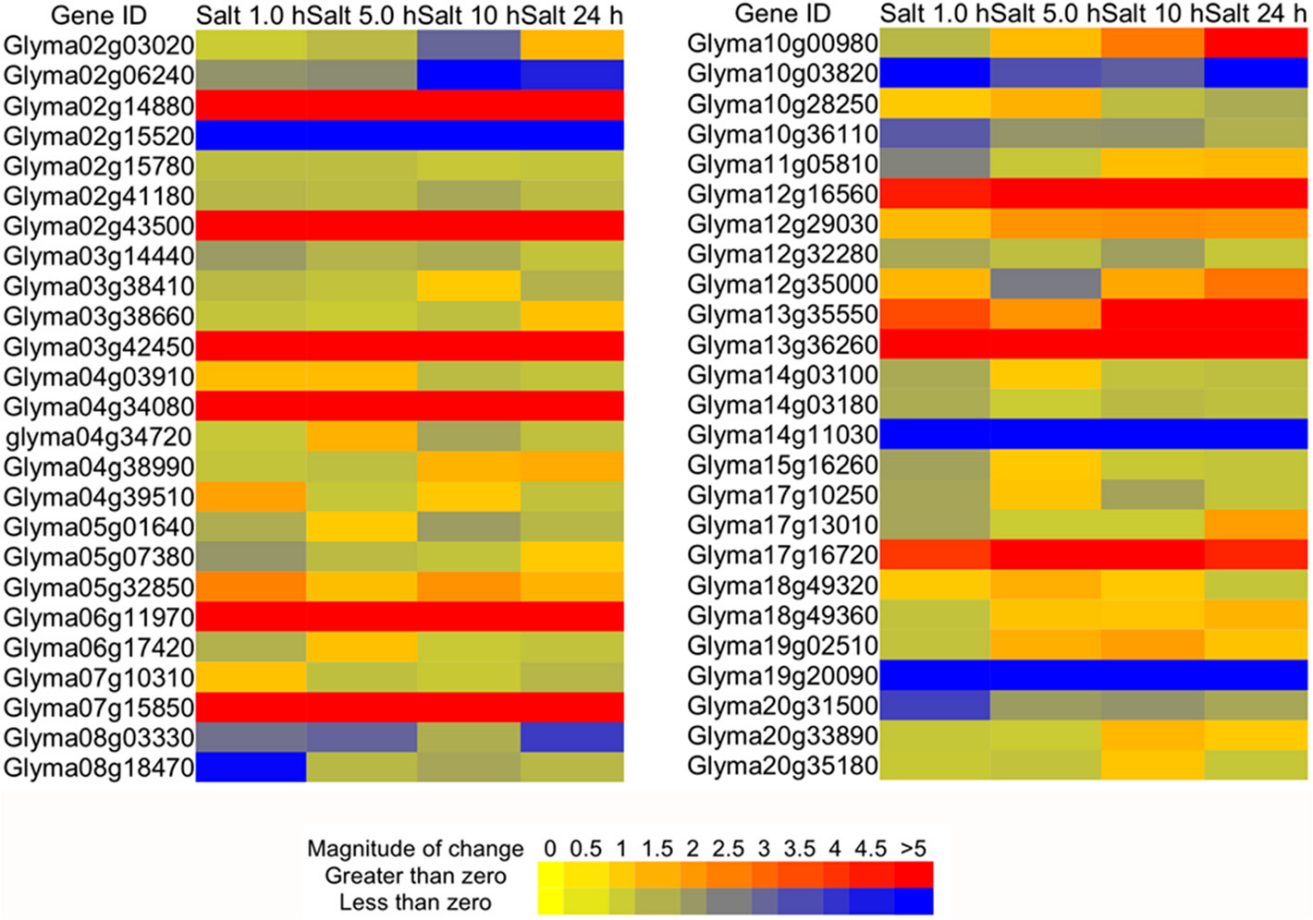
Expression of randomly selected 50 TFs under salt stress conditions for one hour (Salt 1.0 h), five hours (Salt 5.0 h) and ten hours (Salt 10 h), and twenty-four hours (Salt 24 h), respectively

Salinity is another abiotic stress that significantly reduces soybean yield, and plant responses to salt stress and drought are very closely related due to their overlapping mechanisms [28, 29]. Surprisingly, about 80 % of the same 50 TFs were differentially expressed upon salt stress (Fig. 5), suggesting their possible role in salt stress adaption and salinity tolerance. While there was a significant overlap of TFs co-regulated by drought/dehydration/salinity (Figs. 2, 4 and 5), several of them showed opposite expression patterns, such as Glyma19g20090.1, Glyma14g11030.1 and Glyma10g03820.1. Discrepant expressions under these two conditions suggested their distinct roles in response to different stresses.

**Fig. 6:**
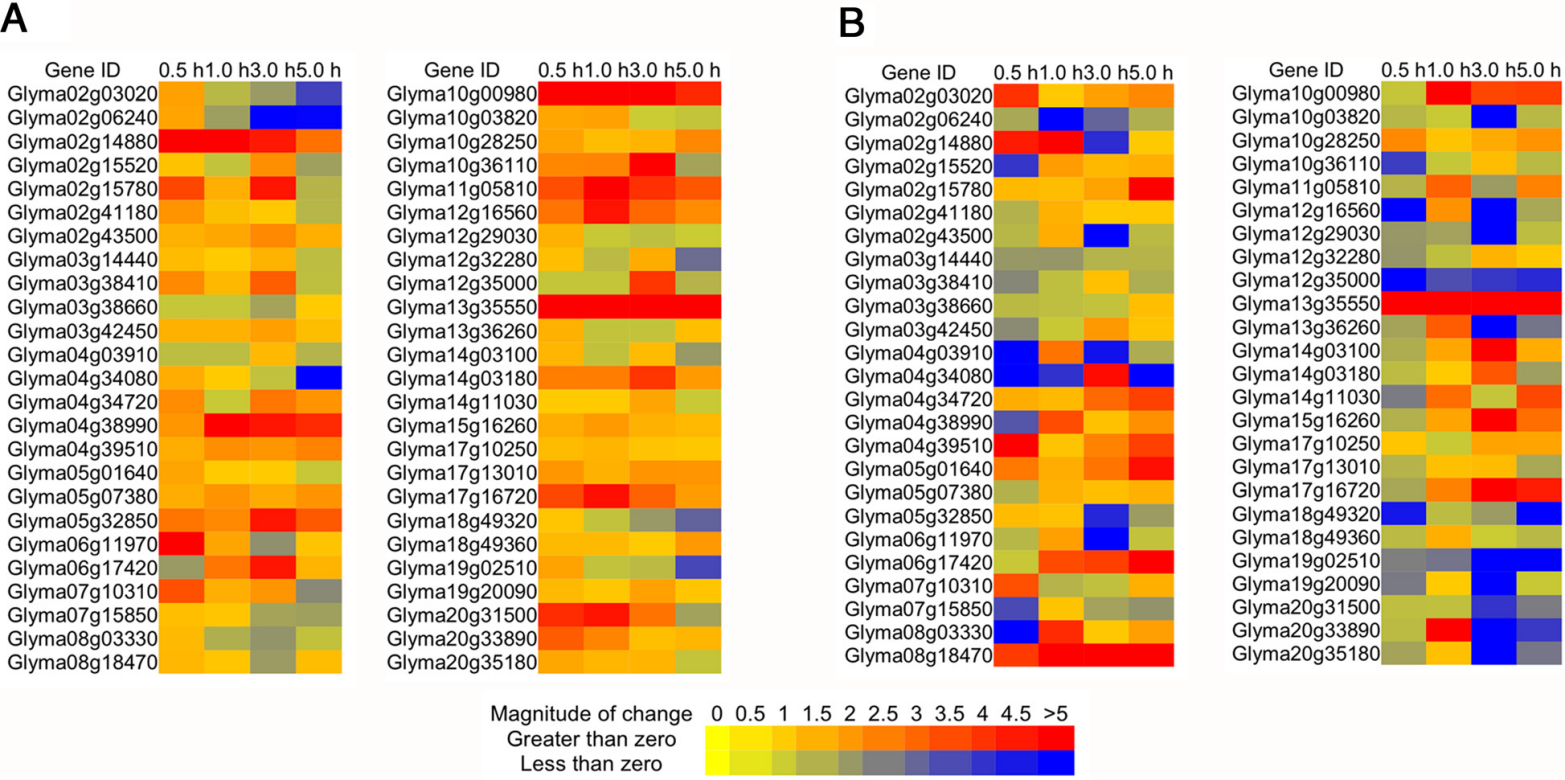
Expression of randomly selected 50 TFs in roots (**a**) and shoots (**b**) under ABA treatment for half hour (0.5 h), one hour (1.0 h), three hours (3.0 h) and five hours (5 h), respectively

The plant hormone abscisic acid (ABA) plays a pivotal role in plant responses to biotic and abiotic stresses [30-32]. When plants are exposed to abiotic stress such as drought and salinity, ABA regulates stomata aperture to limit water loss through transpiration [33]; on the other hand, the localized ABA signaling, by working together with other phytohormones, regulates root growth, especially lateral root growth plasticity [34]. qRT-PCR analysis was performed in order to investigate whether the same set of selected genes are involved in the ABA-dependent signaling pathway (Fig. 6). Expression of the selected genes showed dramatic changes in both roots and shoots under ABA treatments, and 98 % of them showed ≥ 1.5 fold change at one or more of the time points. This result indicated that most of the TFs might function dependently on the ABA signal transduction pathway. More than half of the genes showed a similar expression pattern in roots and shoots, while some other TFs exhibited an opposite pattern in different tissues (such as Glyma05g32850.1 and Glyma20g31500.1), suggesting different roles in shoots and roots (Fig. 6).

### Discovery of cis-regulatory elements (CREs) in soybean TF promoters

Although other alternative mechanisms of gene expression regulation exist, the control of gene transcription via CREs in promoters is still a primary mode of gene expression regulation. Our interest in abiotic stress prompted us to investigate abiotic stress responsive CREs, which may be bound and regulated by other TFs, in the genetic up-stream regions in our soybean TF-ORFeome collection. A total of 21 CREs responsive to abiotic stresses were identified among 200 TF promoters (Additional file 3). However, over-representation analysis did not show any of these CREs significantly enriched in the 1 kb promoters of the 200 TFs.

### Integration of TF-ORFeome resource into SoyKB website

The TF-ORFeome data has been incorporated into SoyKB [15-17]. The data can be directly accessed via the URL [35] after registration. The genes have been linked to the gene card pages (Additional file 5A, B), where users can access other relevant genomic information (Additional file 5C), and multi-omics expression datasets (Additional file 5D, E) available in SoyKB. The motif locations can also be browsed in tabular format or using the graphical visualization Motif Viewer tool. All the results can be downloaded as a CSV file.

### Subcellular localization prediction of cloned TF-ORFs

Protein subcellular localizations are closely linked to their biological functions, and precisely predicting protein subcellular localizations is important for gene function prediction and genome annotation. To maximize the prediction accuracy, results were derived from adopting several publicly available tools [36-39] and carefully analyzed, compared, and combined. Consistent with the putative function of cloned genes in this study as TFs, most of them were predicted to reside in the nucleus (Additional file 3). However, Glyma13g27280.1 was predicted to be localized in the nucleus or chloroplast. Multiple subcellular localizations or altered subcellular localization of proteins are believed to be associated with multiple or altered functions, which have been observed in both mammals and plants [40-44]. Several lines of evidence also showed that nucleus encoded TFs might regulate gene expression, directly or indirectly, in other organelles such as mitochondria and chloroplasts [40, 44]. Furthermore, with the aid of another protein, a TF is able to shuttle dynamically between the nucleus and cytoplasm [45]. It is, therefore, possible that Glyma13g27280.1 functions in both of the organelles. However, experimental investigation is needed for validation of such an assumption.

### Application of soybean TF-ORFeome resources to stresses studies

Since the results presented here are from various comprehensive analyses, plant biologists, especially researchers in the field of abiotic stresses, may find our genomic resources very informative in their search for candidate genes as a starting point. Two examples are given below to demonstrate what function a certain soybean TF may have by putting all data together. Glyma06g17420, one TF from our ORFeome collection, is annotated as a member of the bHLH superfamily, of which 393 members have been *in-silico* characterized in the soybean genome but until now, none have been functionally characterized in terms of drought resistance [46]. Its subcellular localization in the nucleus suggested it might function as a TF (Additional file 3). Its expression was highly up-regulated in shoots upon drought and ABA treatments (Figs. 2 and 6), indicating a role in responding to drought and probably through an ABA dependent pathway. Since it has little similarity with well characterized MYC2 or ICE1, which are positive regulators of drought tolerance [47, 48], exploring the possible novel function of Glyma06g17420 might be interesting.

NAC is one of the largest plant-specific gene families with 152 genes in soybean, and 58 of them are putative stress-responsive genes [49]. Ectopic expression of several of these stress-responsive genes in Arabidopsis enhanced resistance to salinity and freezing [50]. According to our qRT-PCR analysis, Glyma13g35550 (GmNAC101) was highly up-regulated by drought, dehydration, salt and ABA. Recent studies reported that higher expressions of this gene were detected in both shoots and roots of the drought-tolerant cultivar DT51 in comparison with the drought-sensitive cultivar MTD720 under drought conditions [51, 52]. More interestingly, a total of 10 CRE motifs were identified within its 1 kb promoter sequence, indicating that this gene is under complex regulations. All of this evidence suggests that Glyma13g35550 is a potential candidate for in-depth investigation.

## Conclusions

The soybean TF-ORFeome provides a valuable public resource for functional genomics studies, especially in the area of plant abiotic stresses, and will facilitate accelerating the findings in the area of abiotic stresses and in generating crops with enhanced resistance to multiple stresses.

## Methods

### Plant growth, treatments, and tissue collections

Soybean (cv. Williams 82) seedlings were grown in 4-gallon pots containing a mixture of turface and sand (3:1) under the same growth chamber conditions [53]. Drought treatments were initiated by withholding water at the VC stage (stage that cotyledons and unifoliates are fully expanded), while water was provided daily to the well-watered control seedlings. The water potentials for mild and moderate drought were −7 bar and −13 bar, respectively. Dehydration and salt treatments were conducted as previously described [53]. For ABA treatments, two-week-old seedlings were irrigated and sprayed with 200 μM ABA (or a mock solution without ABA as control) and incubated for certain period of times (0.5, 1, 3, and 5 h). After treatment, tissues were harvested and frozen immediately in liquid nitrogen and stored at −80 °C. All samples were collected in biological triplicates.

### RNA isolation and qRT-PCR

Total RNA isolation and qRT-PCR were carried out as described previously [53]. Three biological and two technical replications were conducted in all the qPCR experiments. Gene-specific primers (Additional file 6) for qRT-PCR were designed using Primer3 (version 0.4.0) [54]. The efficacy of primers for qRT-PCR was tested and desirable results were obtained. Soybean Ubiquitin3 gene (Glyma20g27950.1) was used as an internal control for all qRT-PCR analysis.

### Soybean TF-ORF gene cloning

PCR was performed using Phusion high-fidelity DNA polymerase (Thermo Scientific, Pittsburgh PA, USA). PCR products were purified with a gel extraction kit (Epoch Life Sciences, Sugar Land, TX, USA), cloned into pENTR™/D-TOPO® vector or pDONR™/Zeo vector (Invitrogen, Carlsbad, CA, USA), and verified by sequencing using M13 forward and reverse  primers, and additional gene specific primers if necessary. Primers were designed based on sequence information obtained from the Phytozome (v. 9.1) [24].

### TF promoter putative CRE analysis

One thousand base pairs (bps) of the TF promoter sequences retrieved from Phytozome (version 9.1) were subjected to CRE analysis through DNA Pattern Search [55] by referring to the literature [56, 57] and the Stress Responsive Transcription Factor Database (STIFDB) [58].

### TF subcellular localization prediction

Deduced TF protein sequences from experimentally verified ORF sequences were used for predicting TF proteins’ subcellular localization by adopting on-line tools, including WoLF PSORT [36], PlantLoc [37], Cell-PLoc [38], and Euk-mPLoc2.0 [39].

### Availability of supporting data

The data sets supporting the results of this article are included within the article and its additional files.

## Additional files

Additional file 1:
**Fold change of expression of TF genes under water stress.** The expressions (shown as fold change) of soybean TF-ORFeome genes upon drought stress were based on publicly available data [18] and unpublished data (Valliyodan et al.). 5hR1, 5 h of dehydration stress in primary root region 1; 5hR2, 5 h of dehydration stress in primary root region 2; 48hR1, 48 h of dehydration stress in primary root region 1; 48hR2, 48 h of dehydration stress in primary root region 2; SSR, drought stressed roots; SSL, drought stressed leaves; V6L, drought stressed leaves at V6 stage; R2L, drought stressed leaves at R2 stage. The dehydration treatments and soybean primary root region 1 (apical 4 mm) and root region 2 (apical 4–8 mm) were referred to from previous definitions [59]. All of the heat maps in this article were generated using BAR HeatMapper Plus Tool [60]. Additional file 2:
**Validation of expressions of selected soybean TFs in shoots and roots upon drought treatment.** A, genes do not show differential expression upon drought from literature; B, genes show sequence discrepancies compared with genome annotation (Phytozome v9.1). MSL, mild drought stressed shoots; MSR, mild drought stressed roots. qRT-PCR analysis (shown as fold change) of selected soybean TFs for soybean TF-ORFeome construction under mild drought stress (see Methods). Additional file 3:
**Detailed information of soybean TF-ORFs cloned in this study.**
Additional file 4:
**Tissue/organ expression pattern of TF genes.** The expression of soybean TF-ORFeome candidates in seven soybean organs including root, root tip, leaf, shoot apical meristem (SAM), nodule, flower and green pod were based on published RNA-Seq data [26]. The color scale indicates the degree of gene expression levels (yellow, low expression level; red, high expression level). Additional file 5:
**Integration of TF ORFeome information into Soykb.** On the TF-ORFeome data page in SoyKB (A), clicking a gene of interest will lead to its gene card page (B), where the relevant genomic information (C) and multi-omics expression datasets (D, E) can be browsed. Additional file 6:
**Primers used for qRT-PCR in this study.**

## Abbreviations

ABA: Abscisic acid
AP2: APETALA2
bHLH: Basic helix-loop-helix
BR: Brassinosteroids
bZIP: Basic zipper
CDS: Coding sequence
CRE: cis-regulatory element
DRE: Dehydration-responsive element
DREB: Binding to DRE
EREBP: Ethylene-responsive element binding protein
EST: Expressed sequence tag
FPKM: Fragments per kilobase of exon per million fragments mapped
GA: Gibberellin
ICE1: Inducer of CBF expression
JA: Jasmonates
MYB: Myeloblastosis
MYC2: Mylocytomatosis oncogene homolog 2
NAC: NAM/ATAF/CUC
ORF: Open reading frame
PCR: Polymerase chain reaction
RT: Reverse transcription
SA: Salicylic acid
SoyKB: Soybean Knowledge Base
TF: Transcription factor
UTR: Un-translated regions
WGS: Whole genome sequencing
