# Supplementary figures and images for: Soybean transcription factor ORFeome associated with drought resistance: a valuable resource to accelerate research on abiotic stress resistance
Source: BMC Genomics. 2015 Aug 13;16(1):596. doi: 10.1186/s12864-015-1743-6 (PMC4534118; doi:10.1186/s12864-015-1743-6)

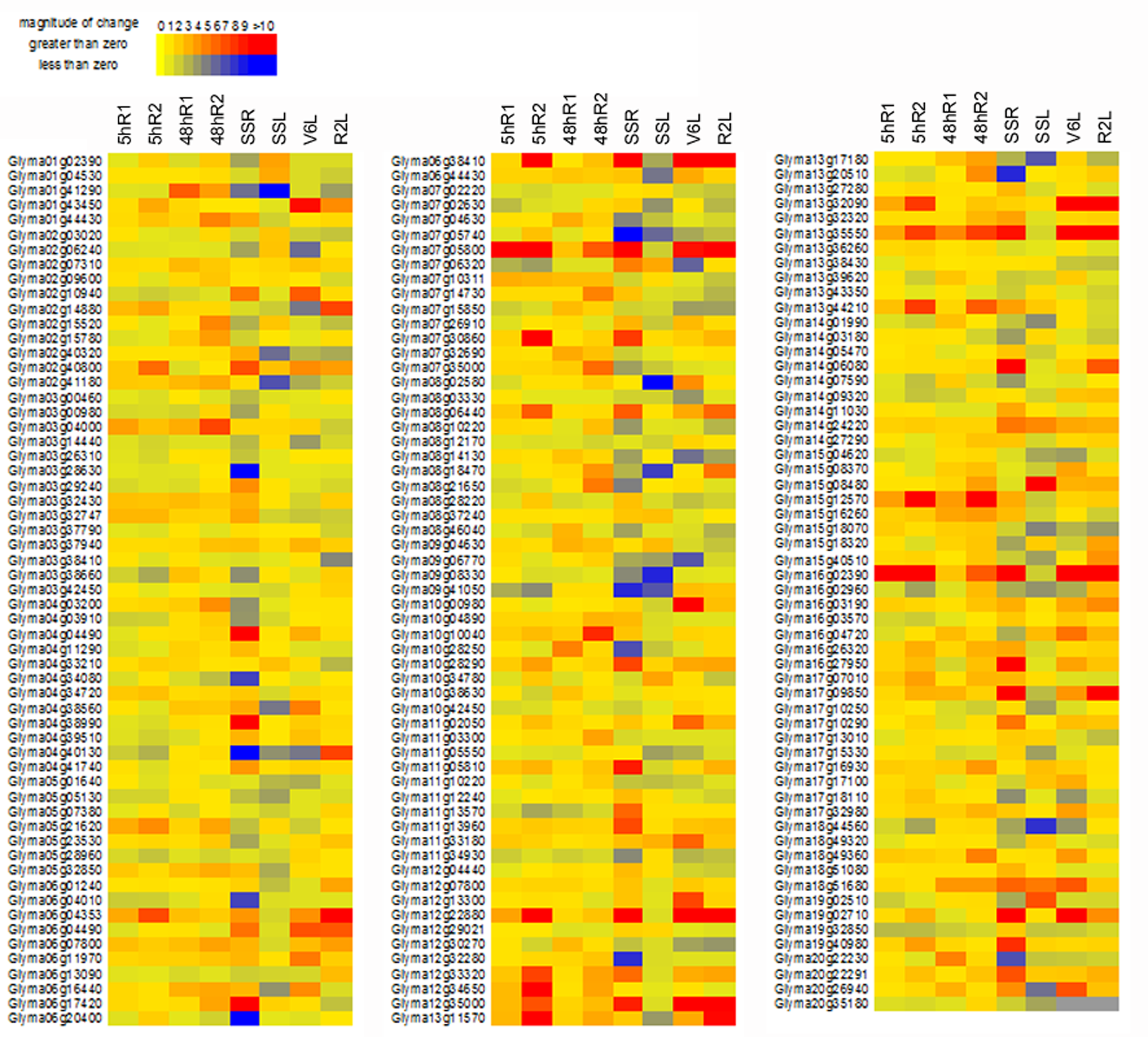

Supplement: Additional file 1: — Fold change of expression of TF genes under water stress. The expressions (shown as fold change) of soybean TF-ORFeome genes upon drought stress were based on publicly available data [18] and unpublished data (Valliyodan et al.). 5hR1, 5 h of dehydration stress in primary root region 1; 5hR2, 5 h of dehydration stress in primary root region 2; 48hR1, 48 h of dehydration stress in primary root region 1; 48hR2, 48 h of dehydration stress in primary root region 2; SSR, drought stressed roots; SSL, drought stressed leaves; V6L, drought stressed leaves at V6 stage; R2L, drought stressed leaves at R2 stage. The dehydration treatments and soybean primary root region 1 (apical 4 mm) and root region 2 (apical 4–8 mm) were referred to from previous definitions [59]. All of the heat maps in this article were generated using BAR HeatMapper Plus Tool [60]. [file 12864_2015_1743_MOESM1_ESM.tiff]

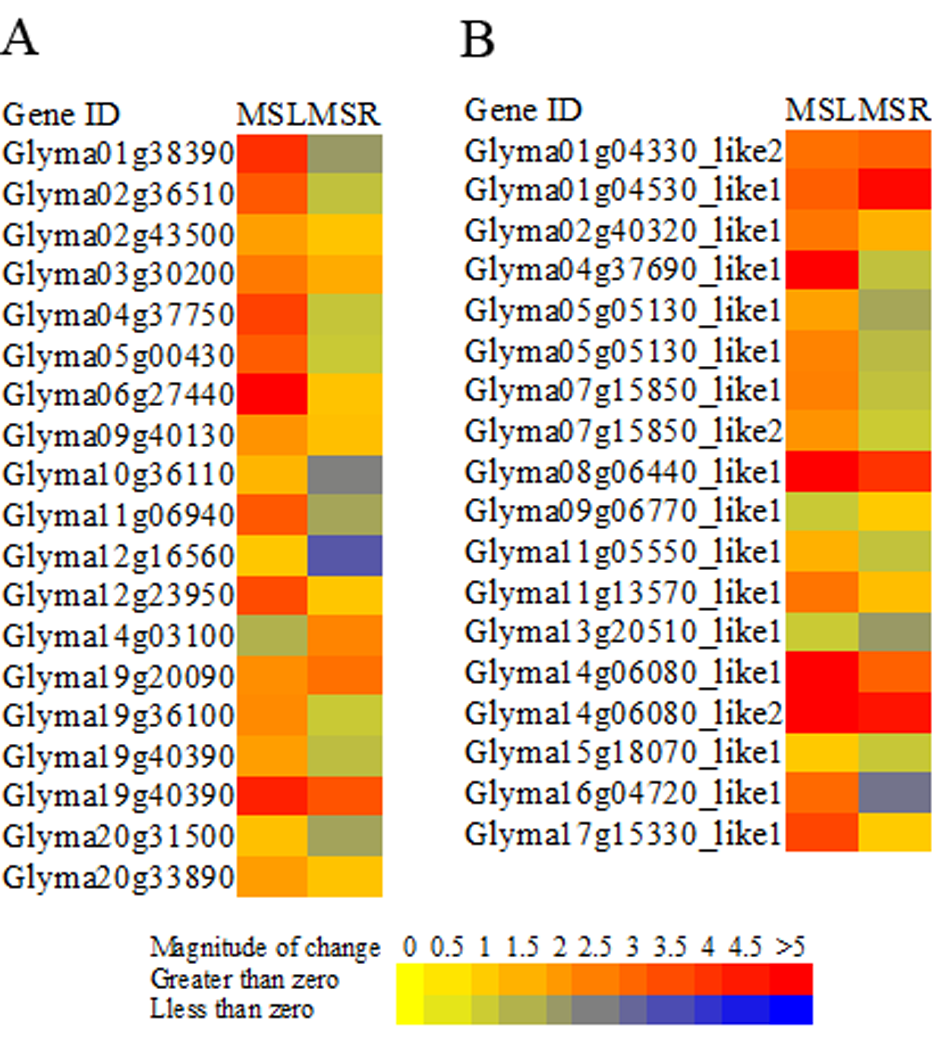

Supplement: Additional file 2: — Validation of expressions of selected soybean TFs in shoots and roots upon drought treatment. A, genes do not show differential expression upon drought from literature; B, genes show sequence discrepancies compared with genome annotation (Phytozome v9.1). MSL, mild drought stressed shoots; MSR, mild drought stressed roots. qRT-PCR analysis (shown as fold change) of selected soybean TFs for soybean TF-ORFeome construction under mild drought stress (see Methods). [file 12864_2015_1743_MOESM2_ESM.tiff]

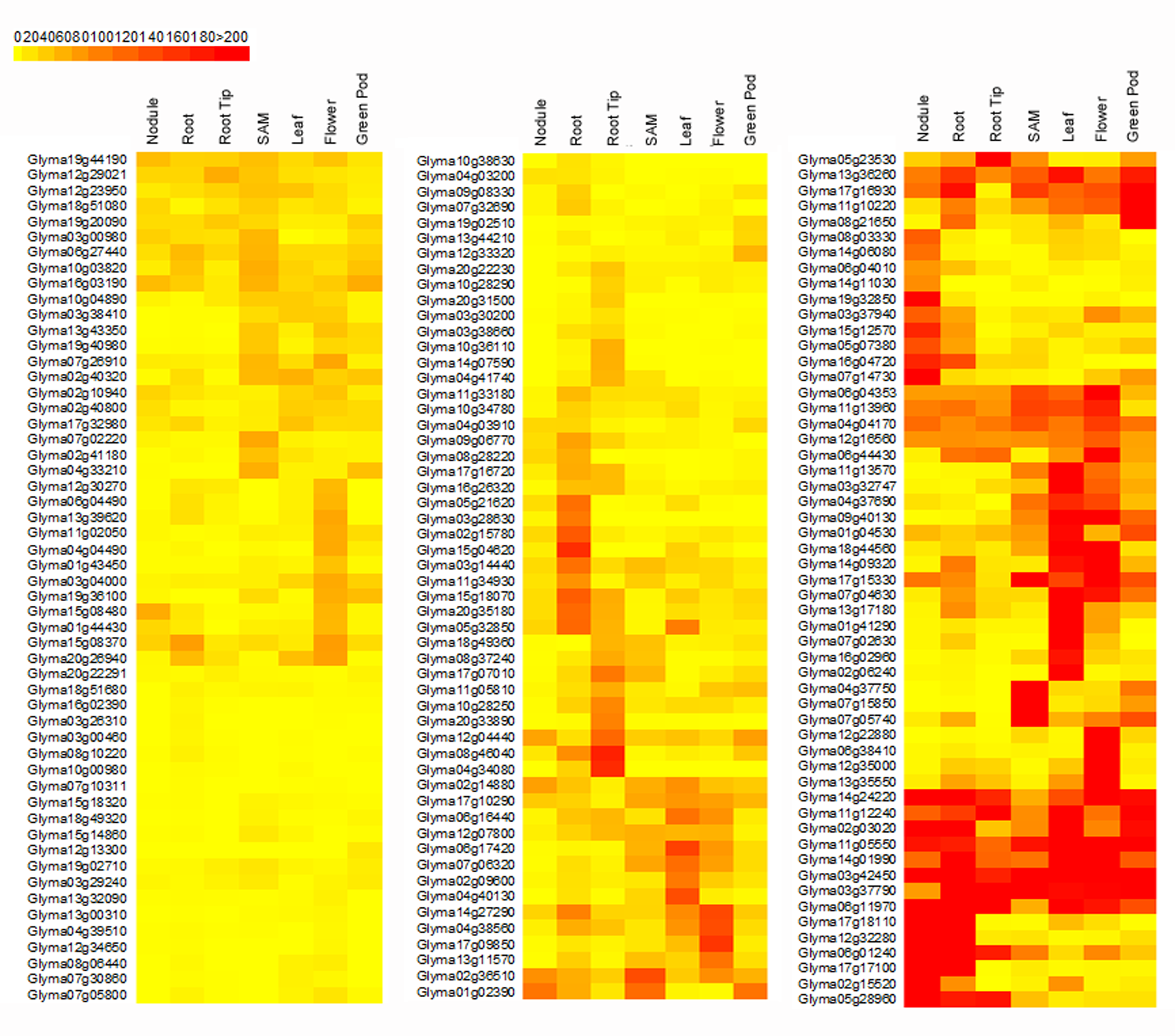

Supplement: Additional file 4: — Tissue/organ expression pattern of TF genes. The expression of soybean TF-ORFeome candidates in seven soybean organs including root, root tip, leaf, shoot apical meristem (SAM), nodule, flower and green pod were based on published RNA-Seq data [26]. The color scale indicates the degree of gene expression levels (yellow, low expression level; red, high expression level). [file 12864_2015_1743_MOESM4_ESM.tiff]
