# Supplementary material for: Soybean transcription factor ORFeome associated with drought resistance: a valuable resource to accelerate research on abiotic stress resistance
Source: BMC Genomics. 2015 Aug 13;16(1):596. doi: 10.1186/s12864-015-1743-6 (PMC4534118; doi:10.1186/s12864-015-1743-6)
Supplement: Additional file 5: — Integration of TF ORFeome information into Soykb. On the TF-ORFeome data page in SoyKB (A), clicking a gene of interest will lead to its gene card page (B), where the relevant genomic information (C) and multi-omics expression datasets (D, E) can be browsed. [file 12864_2015_1743_MOESM5_ESM.pdf]

Additional file 5 Integration of TF ORFeome information into Soykb

On the TF-ORFeome data page in SoyKB (A), clicking a gene of interest will lead you into the gene card page of this gene (B), where you can browse the relevant genomic information (C) and multi-omics expression datasets (D, E).

A

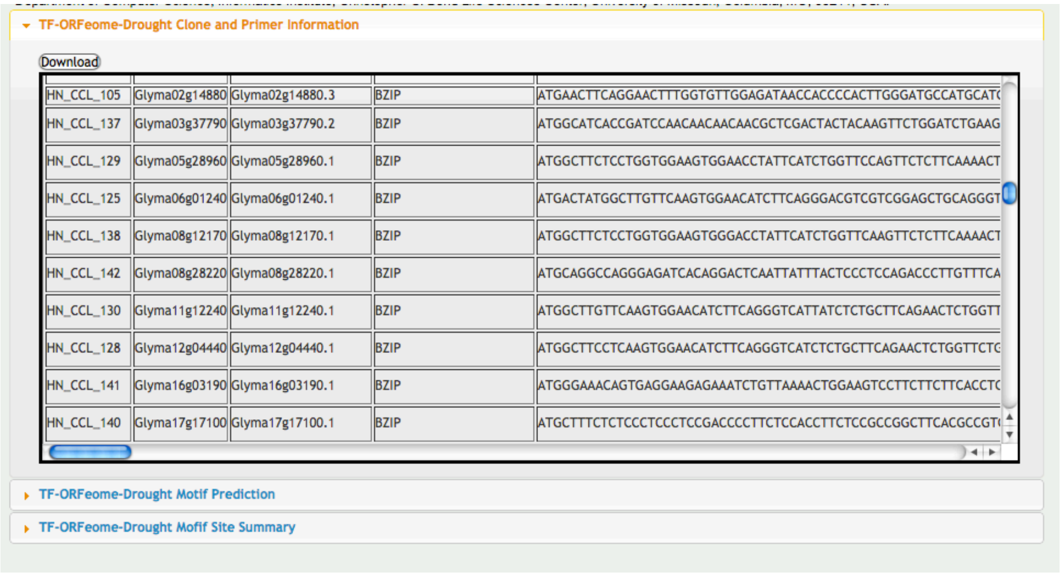

B

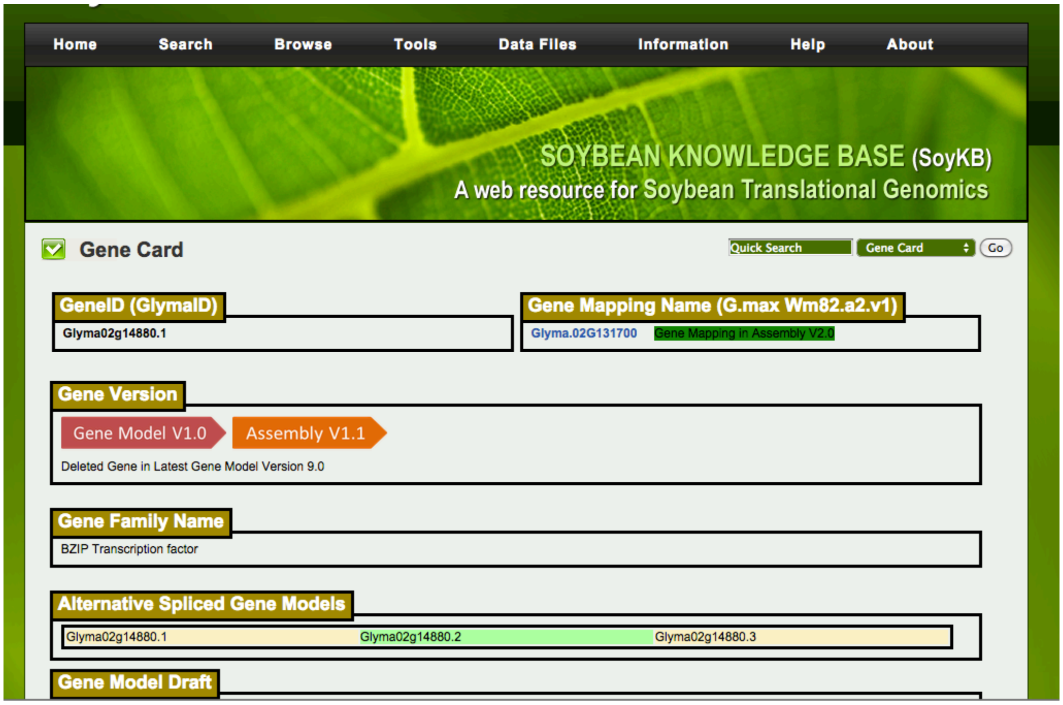

C

Chromosomal Information

		Chromosome	Start	End	Strand	PACID	LetterCode*	Note	GenomeBrowser
Gene									
	mRNA	Gm02	13385858	13388487	-	16248100	E	protein_coding_gene	View
Exons									
	CDS	Gm02	13387345	13388487	-	16248100	E	protein_coding_gene	
	CDS	Gm02	13386434	13386505	-	16248100	E	protein_coding_gene	
	CDS	Gm02	13386304	13386333	-	16248100	E	protein_coding_gene	
	CDS	Gm02	13386132	13386206	-	16248100	E	protein_coding_gene	
UTRs									
	3'-UTR	Gm02	13385858	13386131	-	16248100	E	protein_coding_gene	

*refer to : http://www.phytozome.net/soybean.php

E EST consistent

QTL Traits

Traits	Chromosome	Start	End
FUNGAL	GM02	11820000	14972000
IRRIGATED YIELD NGUYEN LAB	GM02	11465400	23777900
LEAF-STEM	GM02	1773000	22655000
ROOT DRY WEIGHT NGUYEN LAB	GM02	11465400	23777900

SNP

GWAS

Chromosome	Position	Ref C	W01 Beijing4	W02 Donggou16	W03 Wuhai4	W04 Yanjin3	W05 Mengjin1	W06 Jidong5	W07 Fengcheng20	W08 Jixian11	W09 Kaiyuan21	W10 Shuangcheng4	W11 Taiyuan67	W12 Anqing18	W13 Yimeng15	W14 Yimeng19	W15 Yanjir
Gm02	13386705	T	T	T	T	T	T	C	C	C	T	Y	T	T	T	T	T
Gm02	13386995	T	T	T	T	T	T	C	C	C	T	Y	T	T	T	T	T
Gm02	13387071	C	C	Y	C	C	C	T	-	-	C	-	C	C	T	-	T
Gm02	13387211	G	G	G	G	G	G	G	G	G	G	G	G	G	S	C	G
Gm02	13387336	A	A	R	A	A	A	G	G	G	A	R	A	A	R	R	G
Gm02	13388030	T	T	A	T	T	T	-	A	A	T	W	T	T	W	W	A
Gm02	13388464	A	A	M	A	A	A	C	C	C	-	C	A	A	A	A	C

D

Fast Neutron Mutant Data

(special license needed to access the data)

Sequence Evidence

EST - *No data available for this gene ID*

5' RATE - *No data available for this gene ID*

Full Length cDNA - *No data available for this gene ID*

Epigenomics Evidence

Methylation Data - *No data available in this gene.*

Proteomics Evidence

Proteomics[B.japonicum Inoculation] - *No data available in this gene.*

Proteomics[Heat Stress] - *No data available in this gene.*

Proteomics [Public Data] - *No data available in this gene.*

Transcriptomics Evidence

Rna-seq(Solexa/Illumina)

Transcriptomics [Solexa/Illumina_RPKM]

Transcriptomics [Solexa/Illumina_ReadCount] *(special license needed to access the data)*

Microarray

Transcriptomics [Microarray - Leaf and Root] (special license needed to access the data)

Transcriptomics [Microarray - Tissue]

Transcriptomics [Microarray - Stress]

Transcriptomics [Steve-Affy] *(special license needed to access the data)*

Transcriptomics[Melissa] - *No data available for this gene ID*

Oligo

Transcriptomics[Oligo] - *No data available for this gene ID*

CDNA

Transcriptomics[CDNA] - *No data available for this gene ID*

Rna-seq (Trinity)

Transcriptomics[Steve-CD215] - *No data available for this gene ID*

Transcriptomics[Steve-IAC100] - *No data available for this gene ID*

E

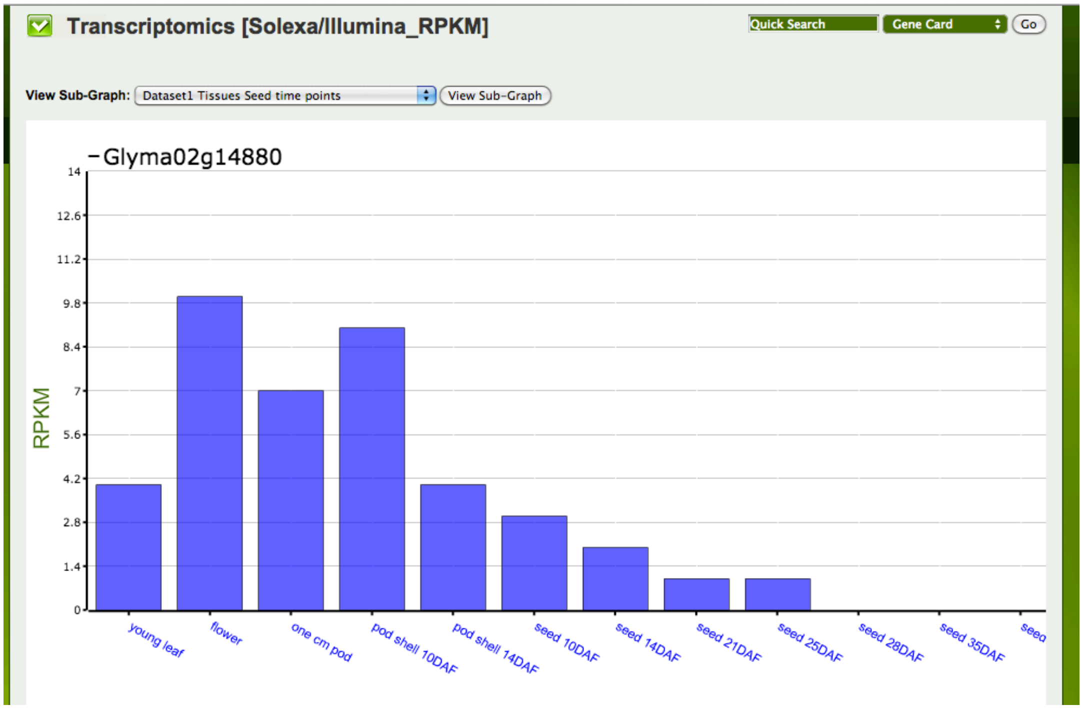

Transcriptomics [Solexa/Illumina_RPKM]
Quick Search
Gene Card
Go
View Sub-Graph:
Dataset1 Tissues Seed time points
View Sub-Graph
Glyma02g14880
RPKM
14
12.6
11.2
9.8
8.4
7
5.6
4.2
2.8
1.4
0
young leaf
flower
one cm pod
pod shell 10DAF
pod shell 14DAF
seed 10DAF
seed 14DAF
seed 21DAF
seed 25DAF
seed 28DAF
seed 35DAF
